# Capitalising on chaos – exploring the impact and future of social media influencer engagement during the early stages of a global pandemic

**DOI:** 10.1177/1329878X20958157

**Published:** 2021-02

**Authors:** Catherine Archer, Katharina Wolf, Joseph Nalloor

**Affiliations:** Murdoch University, Australia; Curtin University, Australia; Murdoch University Dubai, United Arab Emirates

**Keywords:** COVID-19, disaster capitalism, health communication, influence, pandemic, public health, social media, social media influencer

## Abstract

This article examines the role of influencers during the early stages of the COVID-19 pandemic, as well as the impact of the global pandemic on Social Media Influencers’ (SMIs) lifestyle and business model, using the concept of Disaster Capitalism as a springboard for discussion. Worldwide it first appeared that the global pandemic would severely impact SMI sole traders, as income from travel, luxury goods and other ‘lifestyle’ brands dried up. However, we suggest that brands and influencers themselves have pivoted to meet the COVID challenge, with some brands exploiting the opaque influence of these micro-celebrities. We further suggest that while a handful of governments and health organisations have recognised the reach and social capital of SMIs, their potential in health communication has been underutilised. We write this essay as a starting point, raising questions and calling for further research to be conducted to inform the understanding of SMIs’ role and potential as conveyors of public health information.

With the arrival of COVID-19, the question of communication ‘influence’ has been at the forefront of thinking for governments and health authorities worldwide, as they grapple with the best way to convince individuals to protect themselves and the community from the potentially fatal virus, including encouragement to isolate at home. At the same time, the global pandemic has shone a spotlight on the sometimes-questionable influence of so-called social media influencers (SMIs), or ‘micro-celebrities’ (see Senft, 2013). Leading up to COVID-19, SMIs were increasingly engaged by major not-for-profit organisations, governments and, in particular, commercial brands to create and distribute content to audiences for purposeful objectives (Audrezet et al., 2020; McCosker, 2018). Influencers have been identified as being a strategic and powerful avenue for the promotion of products and ideas (Klassen et al., 2018). In 2019, it was predicted that brands were expected to spend $15 billion on influencer engagement globally by 2022 (Schomer, 2019). Given the increasing impact of SMIs, we ask the following questions: how has COVID-19 impacted on the influencer industry, and how have influencers impacted on the spread of information during the crisis?

While this essay can of course not fully answer these questions, it is instead positioned as a starting point for further discussion and future research into the role of SMIs as part of the evolving health pandemic. We examine the impact of the early stages of COVID-19 on the Western, English-speaking influencer industry, and the influence of SMIs on the spread of information through the lens of Disaster Capitalism. Coined by the Canadian author and social activist Naomi Klein in her 2007 work *The Shock Doctrine*, Disaster Capitalism Theory suggests that disasters can be – and indeed frequently are – exploited by capitalist economic interests to grow influence and economic benefits. Originally predominantly focussed on natural disasters, Klein contends that the public’s disorientation following a collective shock – as a result of wars, coups, terrorist attacks, market crashes and natural disasters – is frequently exploited to push through radical capitalist agendas (Klein, 2007; Solis, 2020). While the concept of Disaster Capitalism may mostly apply to large corporations and governments, rather than to individuals, the approach of exploiting the opportunities provided by such a global sense of disorder may suggest that some (from individual entrepreneurs to large-scale companies) will use the resulting collective sense of alarm and disorientation to their benefit and profit.

While Klein refers to the imposition of neoliberal agendas during times of suspended democratic norms, the authors suggest that the lens of Disaster Capitalism can be extended to examine influencers’ actions during one of the largest global crises experienced in modern times: the outbreak of COVID-19 and resulting travel bans, the lock down of borders and emergency measures that have restricted freedom of movement. Although COVID-19 has essentially resulted in a health crisis, the global pandemic has furthermore triggered and exposed multiple other crises, such as crashing stock markets, record unemployment, a lack of infrastructure to manage the demand on health systems and economic recessions around the globe. Furthermore, the health crisis shines a light on aspects of society that might otherwise not be so obvious or taken for granted, such as entrenched social inequalities and social marginalisation (see Dingwall et al., 2013; Lupton, forthcoming). Singapore’s apparent blindness to the risks for its vulnerable migrant worker populations is one obvious example (Sturmer, 2020).

## Influencers: killed off or very much alive?

As countries around the world went into lockdown, businesses wound back their operations and marketing spends were cut extensively, some observers predicted that the social media influencer industry would be all but ‘killed off’ (see e.g. Hamdan, 2020; Tsapovsky, 2020). As the focus shifted from economic prosperity and personal gratification to public health and increasingly community-based engagement, COVID-19 appeared to have resulted in the end of influencer marketing as we know it.

However, as the world adapted to living during a pandemic, it increasingly emerged that many commercially driven influencers would continue to thrive, suggesting that because people are hungry for online content, influencers have become even more important as an avenue for the marketing of ideas and products (see Stephens, 2020). Statistics indicate that social media use has increased dramatically during the lockdown period, with a survey across 30 markets showing engagement increasing 61% over normal usage rates (Holmes, 2020). While some influencers have clearly struggled to adapt to changing market conditions and rapidly contracting commercial opportunities (see Elliott, 2020), others have embraced the global crisis, exploiting public uncertainty and disorientation to build their power, reach and essentially post-COVID marketability and market share (Ewens, 2020).

COVID-19 has impacted every part of our lives and forced many to re-assess priorities. The sheer extent of the virus’ impact has resulted in confusion, fear and uncertainty. On one hand, the public has been bombarded with pandemic-related information and updates, but on the other, an information vacuum has emerged as individuals have been forced to re-assess whose interpretation of the crisis and consequent advice to trust. Some of the traditionally most trusted and relied on sources of information, such as governments, health authorities, and even the World Health Organization (WHO), have been challenged and questioned (see e.g. ABC News, 2020). The WHO has been forced to actively re-build their credibility by contracting dedicated communication counsel and support (McCauley, 2020). Simultaneously, high profile individuals like the US President Donald Trump have publicly endorsed controversial virus treatments like the injection of bleach (BBC News, 2020), which have immediately been challenged by health professionals (Noor, 2020). As a result, large groups of people have ignored critical health advice, as they became to doubt the veracity of information or fell prey to the large volumes of mis- and disinformation that quickly filled any vacuum. As the Edelman Trust Barometer Special Report (2020) on COVID-19 documents, due to a loss in trust in traditional information sources – including governments, NGOs and the media – the public has turned to the private sector for ‘reliable’ advice and guidance.

## Making the most of the new normal

The focus of this article is predominantly on Klein’s (2007) notion of Disaster Capitalism, as opposed to the linked Shock Doctrine strategy, which emphasises the establishment of controversial and questionable policies, as well as the privatisation of goods and services. Nevertheless, we argue that just as private industries spring up to directly profit from large-scale crises, influencers deliberately position themselves to take advantage of the atmosphere of destabilisation for personal gain. Moreover, rather than approaching the current phase of disorientation through a lens of short-term commercial opportunism, SMIs have strategically positioned themselves as trusted information (and entertainment) sources, actively playing a role in deepening inequalities between those caught up in mass redundancies and economic cut backs, and the ‘elite’ who is being looked up to for solutions. SMIs have effectively embraced the neoliberal capitalist system, by rapidly pivoting to benefit from the upheaval.

Immediate commercial opportunities may have at first seemed limited, as the world grappled with the sheer speed and extent in which the virus spread, but influencers have demonstrated their ability to exploit the public’s disorientation by offering a much needed source of escapism, in the form of entertainment (see, e.g. Sparks, 2020) and even free live PE programme for hundreds of thousands of children as they faced months of home schooling (see #PEwithJOE by @thebodycoach) (Guinness World Records, 2020). Some of these initiatives may be framed as a ‘community service’; however, they are arguably the result of deliberate strategic repositioning. Participating influencers know that commercial opportunities will return once the crisis subsides, ultimately enabling them to profit from their increased profile, authority and network. Others have exploited the disaster as an occasion for agenda setting, touting increasingly controversial and often dangerous information, with Australian chef Pete Evans’ promotion of a light device to combat COVID-19 highlighted as a stand-out example (Doherty, 2020). Indeed, influencers have been identified as ‘key distributors’ of COVID-19 misinformation (Waterson, 2020) in their desire to gain attention, build clout and set themselves up for future commercial success.

## The rise of covert selling

It would appear that far from being in decline, the use of SMIs as the conduit to ‘peddle products’ and ideas (sometimes covertly) before and during the pandemic has increasingly extended to industries that face advertising restrictions, including ‘big pharma’ and ‘big tobacco’, as these recognise the selling power of people with large social media followings (Rowell, 2020; Thomas, 2019). For example, during the early stages of the global pandemic British American Tobacco (BAT) began co-opting (some would say high-jacking) universal health messages and hashtags, effectively positioning themselves as public health advocates. These were then placed on branded face masks, which were subsequently handed out, free-of-charge, to SMIs. As Rowell (2020) suggests, BAT’s ‘strategy is to use the pandemic to try and shift their image from vilified industry to trusted health partner’, engaging SMIs to do this in their quest to recruit new users and reposition themselves. BAT’s new mission statement is ‘stimulating the senses of a new adult generation’. The use of covert marketing tactics via SMIs on Instagram is a key tactic, especially given extant advertising restrictions. In return, SMIs get to capitalise on audiences’ disorientation and unique commercial opportunities by strategically positioning themselves and raising their (global) profile. According to the *British Medical Journal*, health and pharmaceutical brands are frequently using SMIs in collaborations that are not clearly declared (Thomas, 2019). COVID-19 appears to have accelerated efforts, as audiences look to SMIs as a source of entertainment and guidance during a time of confusion.

## The capabilities of SMIs: a missed opportunity?

However, while ‘SMIs’ are often thought of as a homologous concept, the label fails to adequately reflect the vast variety of commercialisation styles and arrangements, communication styles and content. Defined by Abidin (2015) as ‘everyday, ordinary internet users who accumulate a relatively large following on blogs and social media through the textual and visual narration of their personal lives and lifestyles, engage with their following in digital and physical spaces, and [who] monetise their following by integrating “advertorials” into their blog or social media posts’, the term social media influencer could now be applied to everyone from nano-influencers with less than 1000 followers on Instagram and subject matter experts, through to politicians such as the US President Donald Trump and celebrities, such as Kim Kardashian. However, the diversity of those on social media with influence is not always reflected in mainstream media, with many articles highlighting SMIs, particularly on Instagram, whose lavish lifestyles have been curtailed due to COVID-19 (see e.g. Bishop, 2020).

Ironically, as influencer engagement has increasingly shifted towards an advertising or promotional model, the potential value of SMIs to promote credible health messages and influence behaviours has been largely ignored by most governments and health authorities. The Australian Government notably ‘burnt its fingers’ in a clumsy foray into influencer marketing for health in 2018. The Federal Health Department paid influencers to promote an initiative to get more young women to engage in physical exercise, using the hashtag #GirlsMakeYourMove (Sweeney, 2018). The campaign also involved mainstream media advertising. While seemingly a laudable initiative, it was uncovered that background checks were lacking. Some of the paid influencers were previously paid by alcohol companies to endorse drinking or had promoted unhealthy weight loss products and regimes (Bennett, 2018), both examples of endorsements that directly contradict the government’s healthy lifestyle message. One influencer had previously posted racist and homophobic comments (Sweeney, 2018). As a result of negative media coverage, in a swift – and some would argue a knee-jerk reaction – the Federal Government announced what now is effectively a ban on any future use of influencers by Australian government departments (Bennett, 2018). Given this history, Australian authorities have generally focussed on health and Government spokespeople as key ‘influencers’ online and in mainstream media, although arguably these may not have the same appeal of their social media counterparts.

One exception to country governments working with influencers to promote COVID-19 health and safety messaging is Finland, which very early into the pandemic classified SMIs as crucial actors to society alongside doctors, bus drivers and grocery store owners (Heikkilä, 2020). In another example, a US not-for-profit group has enlisted influencers to promote COVID-19 health messages. According to a Washington Post report, the National Organising Coalition On Virus Information Distribution – or No COVID project – determines which counties are most vulnerable, and enlists ‘celebrity messengers who have followers there along with trusted local leaders such as physicians, fire chiefs and fifth-grade teachers to spread the word’ (see https://nocovid.us/). The authors suggest that public health communication has in many ways failed to catch up to the online spread of mis- and dis-information during COVID-19. The membership of the Coalition features an impressive list of consultants, former government officials and university researchers. The use of SMIs has been one of their key tactics. On a broader scale, the UN’s own website reports that it has joined forces with a Bolivian rapper to ‘beat COVID-19’, using the increasingly popular platform of TikTok (UN Covid Response, 2020). Again, this is one localised example and whether the rapper has been paid is not clear.

## Influencers beyond COVID

SMIs originally earned their followers’ trust as they were perceived to be authentic and ‘people like us’ (Abidin and Ots, 2016). However, the focus has shifted from content collaboration to something that increasingly resembles a traditional ‘cash for comment’ advertising model. As a result, influencers have essentially commodified their audiences by drawing on neoliberal dynamics to exploit and exacerbate existing differences and inequalities. While some laudable examples of SMIs and celebrities spreading credible, reliable health information do exist, other influencers, desperately seeking new revenue streams, chose to exploit the global pandemic to offer us free market ‘solutions’ to escape the uncertainty and position themselves to further gain post-COVID dollars. In conclusion, we argue that a well-researched and effective health messaging campaign that incorporates SMIs into its tactics could be valuable, but that public health communicators have largely ignored the social capital of (traditional and emerging) influencers at their peril. Examined through a Disaster Capitalism lens, fresh questions emerge in relation to ethical communication and SMIs’ role in society. Future research would benefit from interviews with influencers in different categories and different geographical locations to determine their reactions to the challenge of the pandemic. The slippery categories and shifting terrain for influencers and brands makes this research difficult but necessary. It would also be useful to survey and interview those communication professionals behind brands, who have had their budgets cut as a result of the pandemic, about their views on the future of influencer marketing and engagement.

As the COVID-19 pandemic sweeps across the globe, this article draws on early insights and hence can only ever be positioned as a conversation starter. The authors have used publicly available media reports as the main source of information, so this article has obvious limitations. We suggest this article signals that there is an urgent need to continue research into influencers and their role in society and in particular within the context of health communication, as audiences continue to trust their information often more than that provided by conventional health and government authorities. We believe SMIs’ influence will continue to grow, shaping and being shaped by Disaster Capitalism at play during and beyond the COVID-19 pandemic.

## References

[bibr1-1329878X20958157] ABC News (2020) Donald Trump breaks US ties with WHO, moves to end Hong Kong privileges as coronavirus tensions with China surge. Available at: https://www.abc.net.au/news/2020-05-30/donald-trump-breaks-ties-with-who-over-coronavirus/12303168 (accessed 10 June 2020).

[bibr2-1329878X20958157] Abidin C (2015) Communicative intimacies: influencers and perceived interconnectedness. Ada: A Journal of Gender, New Media, and Technology (8). Available at: https://adanewmedia.org/2015/11/issue8-abidin/ (accessed 28 August 2020).

[bibr3-1329878X20958157] Abidin C Ots M (2016) Influencers tell all? Unravelling authenticity and credibility in a brand scandal. In: Edstrom M Kenyon AT (eds) Blurring the Lines: Market-Driven and Democracy-Driven Freedom of Expression. Goteborg: Nordicom, pp. 153–164.

[bibr4-1329878X20958157] Audrezet A De Kerviler G Moulard JG (2020) Authenticity under threat: when social media influencers need to go beyond self-presentation. Journal of Business Research 117: 557–569.

[bibr5-1329878X20958157] BBC News (2020) Coronavirus: outcry after Trump suggests injecting disinfectant as treatment. BBC News, 24 April. Available at: https://www.bbc.com/news/world-us-canada-52407177 (accessed 1 June 2020).

[bibr6-1329878X20958157] Bennett L (2018) Government bans use of influencers following investigation. AdNews, 17 August. http://www.adnews.com.au/news/government-bans-use-of-influencers-following-investigation#oPPAGuViBsedFr.1499 (accessed 1 June 2020).

[bibr7-1329878X20958157] Bishop K (2020) The pandemic and the influencer: will the lifestyle survive coronavirus? The Guardian Australia, 2 May. Available at: https://www.theguardian.com/media/2020/may/02/influencers-coronavirus-future-income-marketing-lifestyle (accessed 3 June 2020).

[bibr8-1329878X20958157] Dingwall R Hoffman LM Staniland K (2013) Introduction: why a sociology of pandemics? Sociology of Health & Illness 35(2): 167–173. 23278292 10.1111/1467-9566.12019

[bibr9-1329878X20958157] Doherty B (2020) Chef Pete Evans criticised for trying to sell $15,000 light device to fight coronavirus. The Guardian Australia, 10 April. Available at: https://www.theguardian.com/world/2020/apr/10/chef-pete-evans-criticised-for-trying-to-sell-15000-light-device-to-fight-coronavirus (accessed 4 June 2020).

[bibr10-1329878X20958157] Edelman Trust (2020). Edelman trust barometer special report on COVID-19. Available at: https://www.edelman.com/research/edelman-trust-covid-19-demonstrates-essential-role-of-private-sector (accessed 9 June 2020).

[bibr11-1329878X20958157] Elliott H (2020) When the picture isn’t pretty: how influencers are adapting to lockdown social media stars face uncertain futures as multibillion-dollar revenue streams dry up. Bloomberg, 2 April. Available at: https://www.bloomberg.com/news/articles/2020-04-01/how-social-media-influencers-are-affected-by-coronavirus-shutdown (accessed 8 June 2020).

[bibr12-1329878X20958157] Ewens H (2020) Coronavirus has battered the influencer market: but some are still pulling in work brands are yanking deals and sponsorship is drying up, but some social media stars are still keeping the faith. Vice, 17 April. Available at: https://www.vice.com/en_au/article/5dmvzz/coronavirus-social-media-influencers-effect (accessed 7 June 2020).

[bibr13-1329878X20958157] Guinness World Records (2020) Joe Wicks’ PE with Joe smashes YouTube livestream record. Guinness World Records, 14 April. Available at: https://www.guinnessworldrecords.com/news/2020/4/joe-wicks-pe-with-joe-smashes-youtube-livestream-record-614934 (accessed 5 August 2020).

[bibr14-1329878X20958157] Hamdan L (2020) Has Covid-19 killed off influencers? Arabian Business, 1 April. Available at: https://www.arabianbusiness.com/media/444157-has-covid-19-killed-off-influencers (accessed 2 June 2020).

[bibr15-1329878X20958157] Heikkilä M (2020) Finland taps social media influencers during coronavirus crisis The Nordic country is hoping to harness their networks to get people to #FlattenTheCurve. Politico, 2 April. Available at: https://www.politico.eu/article/finland-taps-influencers-as-critical-actors-amid-coronavirus-pandemic/amp/ (accessed 2 June 2020).

[bibr16-1329878X20958157] Holmes R (2020) Is COVID-19 social media’s levelling up moment? Forbes, 24 April. Available at: https://www.forbes.com/sites/ryanholmes/2020/04/24/is-covid-19-social-medias-levelling-up-moment/#3f4ab3676c60 (accessed 2 June 2020).

[bibr17-1329878X20958157] Klassen KM Borleis ES Brennan L , et al. (2018) What people ‘like’: analysis of social media strategies used by food industry brands, lifestyle brands, and health promotion organizations on Facebook and Instagram. Journal of Medical Internet Research 20(6): e10227. 29903694 10.2196/10227 PMC6024098

[bibr18-1329878X20958157] Klein N (2007) The Shock Doctrine: The Rise of Disaster Capitalism. New York: Metropolitan Books.

[bibr19-1329878X20958157] Lupton D (forthcoming) Contextualising COVID-19: sociocultural perspectives on contagion. In: Lupton D Willis K (eds) The Coronavirus Crisis: Social Perspectives. London: Routledge, pp. 1–10.

[bibr20-1329878X20958157] McCauley K (2020) H+K works to bolster WHO’s credibility: O’Dwyers the inside news of PR & marketing communications. Available at: https://www.odwyerpr.com/story/public/14595/2020-07-17/hk-works-bolster-whos-credibility.html (accessed 10 August 2020).

[bibr21-1329878X20958157] McCosker A (2018) Engaging mental health online: insights from *beyondblue*’s forum influencers. New Media & Society 20(12): 4748–4764.

[bibr22-1329878X20958157] Noor P (2020) ‘Please don’t inject bleach’: Trump’s wild coronavirus claims prompt disbelief. The Guardian, 25 April. Available at: https://www.theguardian.com/us-news/2020/apr/24/trump-disinfectant-bleach-coronavirus-claims-reaction (accessed 3 June 2020).

[bibr23-1329878X20958157] Rowell A (2020) Coronavirus: big tobacco sees an opportunity in the pandemic. The Conversation, 14 May. Available at: https://theconversation.com/coronavirus-big-tobacco-sees-an-opportunity-in-the-pandemic-138188 (accessed 3 June 2020).

[bibr24-1329878X20958157] Schomer A (2019) Influencer marketing: state of the social media influencer market in 2020. Business Insider, 18 December. Available at: https://www.businessinsider.com/influencer-marketing-report?r=AU&IR=T (accessed 4 August 2020).

[bibr25-1329878X20958157] Senft TM (2013) Microcelebrity and the branded self. In: Hartley J Burgess J Bruns A (eds) A Companion to New Media Dynamics. Chichester: Wiley Blackwell, pp. 346–354.

[bibr26-1329878X20958157] Solis M (2020) Coronavirus is the perfect disaster for ‘disaster capitalism’ Naomi Klein explains how governments and the global elite will exploit a pandemic. Vice, 16 March. Available at: https://www.vice.com/en_au/article/5dmqyk/naomi-klein-interview-on-coronavirus-and-disaster-capitalism-shock-doctrine (accessed 30 May 2020).

[bibr27-1329878X20958157] Sparks H (2020) Bozo Instagram influencers are exploiting coronavirus for clout. New York Post, 31 January. Available at: https://nypost.com/2020/01/31/bozo-instagram-influencers-are-exploiting-coronavirus-for-clout/ (accessed 31 May 2020).

[bibr28-1329878X20958157] Stephens L (2020) Why influencer marketing will win after COVID-19. AdNews, 9 April. Available at: https://www.adnews.com.au/opinion/why-influencer-marketing-will-win-after-covid-19 (accessed 20 May 2020).

[bibr29-1329878X20958157] Sturmer J (2020) Singapore was the envy of the world when it came to coronavirus, but then the second wave hit. ABC News, 25 April. Available at: https://www.abc.net.au/news/2020-04-25/singapore-hoped-they-contained-coronavirus-but-second-wave-hit/12172446 (accessed 20 May 2019).

[bibr30-1329878X20958157] Sweeney L (2018) Health Minister announces urgent investigation into taxpayer-funded campaign working with Instagram influencers. ABC News, 20 July. Available at: https://www.abc.net.au/news/2018-07-20/health-department-investigating-instgram-influencer-campaign/10016712 (accessed 20 May 2019).

[bibr31-1329878X20958157] Thomas K (2019) Key opinion leaders supercharged by the internet: paid doctor and patient influencers on social media. BMJ 365: l2336. Available at: https://www.bmj.com/content/365/bmj.l2336 (accessed 3 June 2020). 31152003 10.1136/bmj.l2336

[bibr32-1329878X20958157] Tsapovsky F (2020) Could the coronavirus kill influencer culture? In the middle of a pandemic, how influential can Instagram stars really be? Wired, 14 April. Available at: https://www.wired.com/story/coronavirus-covid-19-influencers/ (accessed 3 June 2020).

[bibr33-1329878X20958157] UN Covid Response (2020) Bolivian rapper drops beats to beat COVID-19. Available at: https://www.un.org/en/coronavirus/un-joins-forces-rapper-bolivia-beat-covid-19 (accessed 3 May 2020).

[bibr34-1329878X20958157] Waterson J (2020) Influencers among ‘key distributors’ of coronavirus misinformation. The Guardian, 9 April. Available at: https://www.theguardian.com/media/2020/apr/08/influencers-being-key-distributors-of-coronavirus-fake-news (accessed 31 May 2020).

